# Ultrastructure of the Bovine Testis in Cattle (*Bos taurus*): New View

**DOI:** 10.3390/ani14121777

**Published:** 2024-06-13

**Authors:** Katarzyna Michałek, Marta Grabowska, Patrycja Oberska, Dariusz Gączarzewicz, Andrzej Syczewski, Septimiu Cassian Tripon, Lucian Barbu-Tudoran, Maria Suciu

**Affiliations:** 1 Department of Physiology, Cytobiology and Proteomics, West Pomeranian University of Technology in Szczecin, Klemensa Janickiego 29, 71-270 Szczecin, Poland; patrycja.oberska@zut.edu.pl; 2 Department of Histology and Developmental Biology, Pomeranian Medical University, Żołnierska 48, 71-210 Szczecin, Poland; marta.grabowska@pum.edu.pl; 3 Department of Animal Reproduction, Biotechnology and Environmental Hygiene, West Pomeranian University of Technology in Szczecin, Klemensa Janickiego 29, 71-270 Szczecin, Poland; dariusz.gaczarzewicz@zut.edu.pl; 4 Genetic and Animal Husbandry, Tomasza 40, 72-006 Szczecin, Poland; biuro@bydlo-as.pl; 5 Electron Microscopy Centre, Faculty of Biology and Geology, Babeș-Bolyai University, 44 Republicii St., 400015 Cluj-Napoca, Romania; septimiu.tripon@ubbcluj.ro (S.C.T.); lucian.barbu@ubbcluj.ro (L.B.-T.); suciu.maria@ubbcluj.ro (M.S.)

**Keywords:** TEM, seminiferous tubule, Sertoli cells, spermatogonia, spermatocyte, development

## Abstract

**Simple Summary:**

The detailed analysis of the structure of individual organs is necessary to understand the processes occurring within them. The complicated nature of male reproductive processes and the still insufficient knowledge of the structure of the male reproductive system prompted us to undertake the present research. In this study, using transmission electron microscopy (TEM) and histology staining, testes of sexually immature calves and reproductive bulls of the Polish Holstein-Friesian Black-and-White breed were analyzed. The obtained results were summarized and compared with previously published reports. The present manuscript presents complementary information on ultrastructure of seminiferous epithelium and interstitial tissue in cattle and provides an essential morphological background for further applied research.

**Abstract:**

The purpose of this study was to analyze the ultrastructure of the testes of sexually immature calves and reproductive bulls of the Polish Holstein-Friesian Black-and-White breed. Utilizing TEM, this study identified three distinct stages of seminiferous tubule development in calves, characterized by varying shapes, distributions, and arrangements of individual cells. In immature animals, early developing spermatocytes, prespermatogonia, and pre-Sertoli cells were observed within the seminiferous tubules. In sexually mature bulls, all cells of the spermatogenic series were observed, situated on a thin, multilayered basal lamina, which forms characteristic undulations. An abundant smooth endoplasmic reticulum was observed in the cytoplasm of spermatogonia in both groups of animals, forming characteristic membranous swirls. In adult bulls, spermatogonia maintain contact with each other through numerous cytoplasmic bridges and cell connections, forming small spaces with visible microvilli between them. The ultrastructural analysis facilitated the identification of morphological changes occurring during the maturation of pre-Sertoli cells, transitioning from a large euchromatic nucleus to a nucleus in which the formation of characteristic vesicles and tubules could be observed. It should also be emphasized that two types of Sertoli cells, namely dark and light electron-dense cells, can be found in cattle. These cells differ from each other, indicating that they may perform different functions. The widespread recognition of the presence of two types of Sertoli cells in cattle will undoubtedly contribute to a better understanding of the processes occurring within the testes and provide a basis for further research in this area.

## 1. Introduction

The global increase in male infertility observed in recent years, both in humans and animals, has prompted the search for new solutions enabling the precise determination of male reproductive potential [1]. It can be very difficult or even impossible to define the probable cause of poor fertilization, especially in males with normal semen parameters and without abnormalities in both the anatomical and functional structure of the reproductive organs [2,3]. Hence, it has long been noted that the current standards and procedures are not sufficient to fully define male reproductive potential and the causes preventing successful fertilization. Searching for new indicators and introducing new procedures is not possible without simultaneously broadening the knowledge on the structure and functioning of the reproductive organs. The modern currently used tools, including transmission electron microscopy (TEM), provide the opportunity to take a “deeper” look at the structure of male reproductive tract and “track” changes not only during its growth and development, but also under the influence of various factors and pathophysiological conditions. The improvement of reproductive methods in both animals and humans is inevitably linked to the advancement of knowledge in this area. The ultrastructure of the male reproductive organs is fairly well described in laboratory animals. Unfortunately, there are few such data concerning livestock, including cattle, in the available literature. The information published to date has focused on a detailed morphological analysis of spermatozoa and the seminiferous epithelium cycle in this animal species, with many studies published back in the 1980s. The available data on the analysis of the ultrastructure of male reproductive organs in cattle mainly concern sexually mature bulls [4,5,6,7,8]. Thus far, the literature data in calves mainly include histological analyses using a light microscope [9,10,11,12]. Among the sparse studies on ultrastructure alterations during development, works describing bovine Leydig cells [13] and germ cells from embryonic life to puberty can be found [8,14,15]. It is noteworthy that these are still single works that constitute one source of information on this subject in this animal species. In view of the still insufficient knowledge about the detailed structure of male reproductive organs in cattle and the growing needs in the field of modern biotechnology of livestock reproduction, this study was conducted to search for differences in the ultrastructure of reproductive testis between sexually immature calves and bulls of high breeding value selected for reproduction using TEM. This work presents an analysis of the testis ultrastructure in both groups of discussed animals. The present observations not only complement information on the ultrastructure of the seminiferous epithelium and interstitial tissue in cattle, but also provide an essential morphological background for further applied research.

## 2. Materials and Methods

The experiment was conducted on males of the Polish Holstein-Friesian Black-and-White breed. For this study, we selected animals in a good physical condition with no apparent defects of the genitalia. The research material consisted of testes collected from two groups of animals (previously described in detail by Oberska et al. [16]). The first group consisted of sexually immature calves from 15 to 20 weeks of age (*n* = 6). The second research group included sexually mature bulls aged 3–4 years (*n* = 6), selected by the Polish Animal Breeding and Insemination Center and intended for slaughter. The reproductive organs of both groups of animals were collected during routine slaughter at a local abattoir. After slaughter, the left male reproductive organs were immediately removed. A representative part of each testis was carefully dissected, rinsed twice in cooled (4 °C) 0.9% NaCl to remove excess blood and fixed in a solution of 2.5% glutaraldehyde in 0.1 M PBS for 2 h at 4 °C. After prefixation, the specimens were washed three times with PBS (3 × 15 min at 4 °C) and then fixed again in 2% OsO_4_ in PBS solution for 1.5 h at 4 °C under the exhaustion hood. Samples were washed in PBS (3 × 15 min at 4 °C) and dehydrated in increased concentrations of acetone to water solutions (30%, 50%, 70%, 90%, 100%, for 15 min each step). The last 100% acetone dehydration step was repeated 3 times for 30 min. Dehydrated samples were then embedded in Epon 812 resin, gradually, using Epon in acetone solutions at ratios of 1:3, 1:2; 1:1, 2:1, and 3:1 (1 h each step)m alogn with pure Epon (overnight). Embedded samples were placed in plastic capsules and left to cure at 60 °C for 2–3 days until hard (polymerized) [17]. Capsules were then cut off, the hardened samples were trimmed and sectioned using Leica UC7 Ultramicrotome, obtaining 150 nm and 50–90 nm thick sections. These sections were collected on 150 mesh Cu grids and stained with uranyl acetate and lead citrate solutions. Images were collected using a MegaView III digital camera in Jeol JEM 1010 TEM (JEOL, Tokyo, Japan) or on a Hitachi SU2700 STEM (Hitachi High-Tech, Tokyo, Japan) using the TEM mode. The 150 nm thick sections were stained with Toluidine blue for histological analysis of the same sections to be imaged in TEM. Sections were collected on a drop of water on glass slides and left to dry. Afterwards, sections were covered with Toluidine blue stain and heated over a flame for 10–15 s. The stain was washed away and slides were left to dry. Images were taken using a light microscope at 40× and 100× (oil immersion) (Olympus B43, Hamburg, Germany). The thinner sections (50–90 nm) were collected on 150 mesh Cu grids and stained with uranyl acetate and lead citrate solutions. Images were collected using a MegaView III digital camera in Jeol JEM 1010 TEM or on a Hitachi SU2700 STEM using the TEM mode.

## 3. Results

### 3.1. Seminiferous Tubules and Intratubular Compartment in Calves

**Figure 1:**
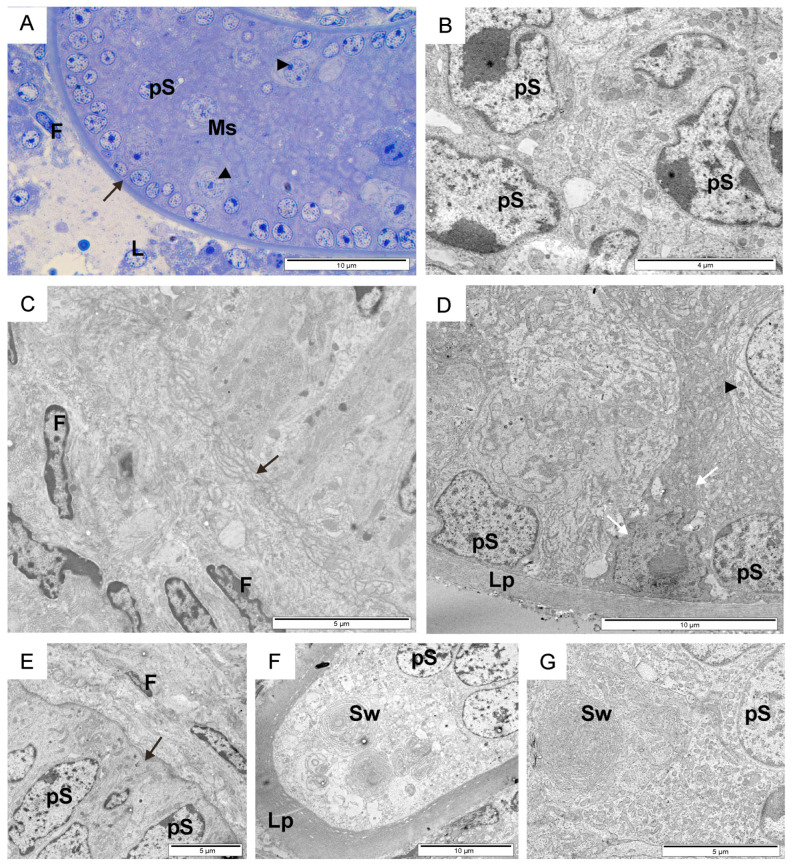
Overview of the seminiferous tubules of calves. Toluidine blue-stained section (**A**). Transmission electron micrographs of testicular sections (**B**–**G**). An early-developing seminiferous tubule (**B**), and the second (**C**,**E**) and third (**D**,**F**,**G**) stages of seminiferous tubule development. Black arrow, basal lamina; black arrowhead, pre-Sertoli cell; F, fibroblast; L, Leydig cell; Lp, lamina propria; Ms, mesenchymal substance; pS, pre-spermatogonia; Sw, membranous swirls; white arrows, support cell.

In calves, within the seminiferous tubules, early-developing spermatocytes, prespermatogonia, and light and dark pre-Sertoli cells were observed, separated from the intertubular space by a thin or thick lamina propria (Figure 1A–G). The ultrastructural analysis of the testicular tissue and evaluation parameters such as the lumen and diameter of the seminiferous tubules, the thickness of the basal lamina, the shape and type of cells lining the tubule, their arrangement, and the distribution relative to the basal lamina allowed for the identification of three distinct developmental stages. The first stage involved the early development of the seminiferous tubule, with a diameter of approximately 50–60 µm. These tubules lacked a visible lumen, and their center was filled with a mesenchymal substance. The interior of the tubules was lined with tightly packed pre-Sertoli cells and prespermatogonia (Figure 1B). The lamina propria consisted of one to two thin layers of electron-dense material, each with a thickness of 0.2–1 µm. In the second stage of development, the diameter of the seminiferous tubules was larger, at approximately 100 µm. Their progressive lumenization was observed. Numerous tightly packed prespermatogonia and early-developing spermatocytes were visible in the lumen of the seminiferous tubules. Pre-Sertoli cells were less frequently observed within them. The basal lamina had an intricate filigree-like layering of 5–10 strata (Figure 1C,E). The seminiferous tubules of the examined calves in the third stage of development had a thick (2–6 µm) laminated basal lamina consisting of three to five layers and shallow spaces between them (Figure 1F). They were lined with loosely arranged cells, among which pre-Sertoli cells, scattered darker supporting cells, and pre-spermatogonia with large membranous swirls were visible (Figure 1D,G). Pre-spermatogonia in the early-developing seminiferous tubules had an ovoidal–polygonal cell shape with a compact ovoid nucleus in the cell center and one to two nucleoli (Figure 1B). In the second developmental stage, these cells were long and thin with an elongated and indented nucleus located near the base of the cell. The nuclei contained one to two large nucleoli and thicker heterochromatin near the nuclear envelope (Figure 1E). A large portion of the cytoplasm filled with organelles (mitochondria and vesicles) was located towards the lumen of the seminiferous tubule. In this same region, the cells were tightly connected to their neighbors by a desmosome-like structure and were in direct contact with the basal lamina. In the early developmental stage, pre-spermatogonia formed two to six pseudostratified layers, while in the second stage, they developed two to three pseudostratified layers. In the third stage, pre-spermatogonia were larger, loosely packed, with a large amount of cytoplasm filled with the aforementioned membranous swirls of smooth endoplasmic reticulum and vesicles (Figure 3D,E). Sometimes, they came into direct contact with the lamina priopria (Figure 3C). A thin layer of cytoplasm was visible between them.

**Figure 2:**
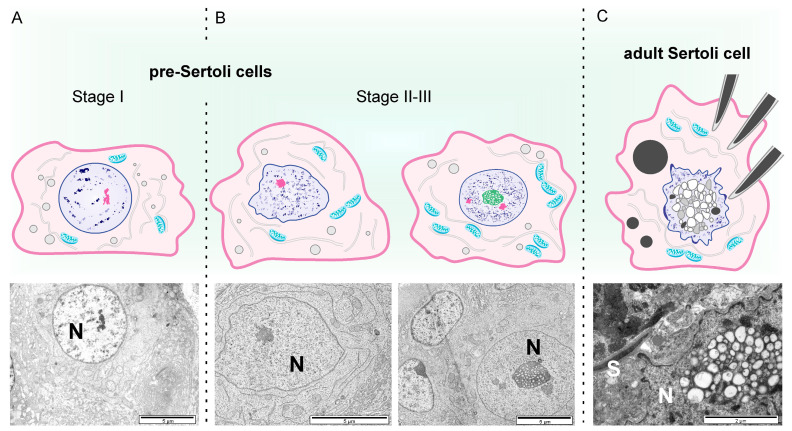
Schematic illustration of bovine pre-Sertoli cell in the I (**A**) and II-III (**B**) stages of seminiferous tubule development. Adult bovine Sertoli cell (**C**). N, nucleus of pre-Sertoli or adult Sertoli cells; S, spermatid.

Two types of supporting cells, light and dark electron-dense cells, were observed in the examined calves. In the early-developing seminiferous tubules, light pre-Sertoli cells appeared as electron-transparent cells, with a basal plasmalemma measuring 5–10 µm, and having a cuboidal or sinuous shape (Figure 2A). Light pre-Sertoli cells can be easily recognized by their large euchromatic nucleus, which is much larger than the surrounding cells, located close to the seminiferous tubule margins. They have a light, apparently empty cytoplasm containing only a few mitochondria, smooth endoplasmic reticulum membranes, and vesicles. In the third stage of seminiferous tubule development, the nucleus of light supporting cells contained one large central nucleolus. Sometimes, heterochromatin arranged in a network of strands, forming characteristic vesicles between them, was visible (Figure 2B).

**Figure 3:**
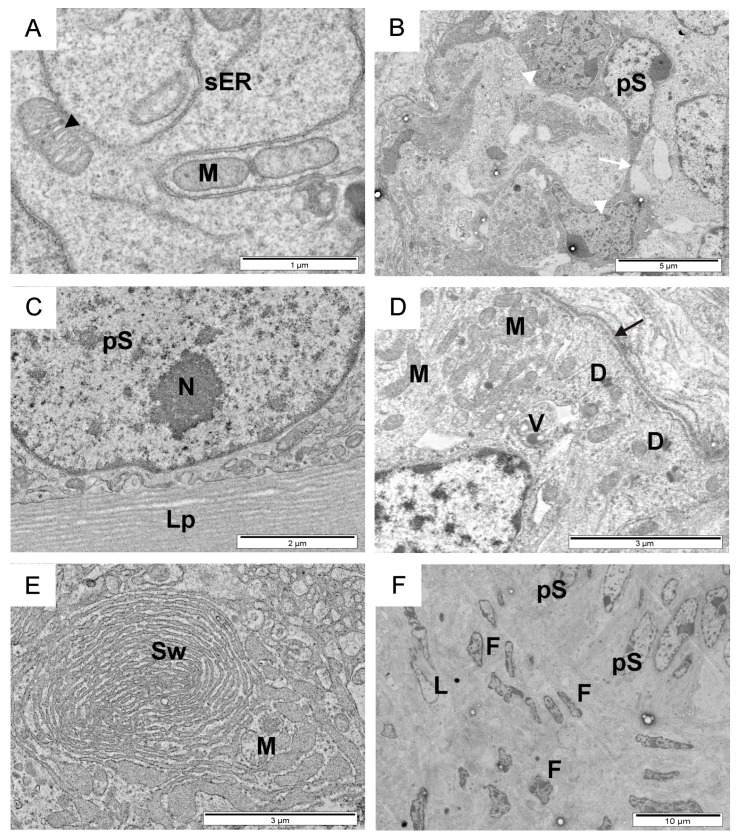
Transmission electron micrographs of the cytoplasmic region of pre-Sertoli cells (**A**), dark support cells within the seminiferous tubule (**B**), direct contact of pre-spermatogonia with the basal lamina (**C**), mitochondria and vesicles, including desmosomes (**D**), membranous swirls in the cytoplasm of pre-spermatogonia (**E**), and the intratubular space with a fragment of seminiferous tubules (**F**) in calves. Black arrow, basal lamina; black arrowhead, contacts between the mitochondria and smooth endoplasmic reticulum; D, desmosomes; F, fibroblast; M, mitochondria; N, nucleoli; L, Leydig cell; Lp, lamina propria; pS, pre-spermatogonia; sER, smooth endoplasmic reticulum; Sw, membranous swirls; white arrow, contact between two support cells; white arrowhead, support cells; V, vesicles.

Numerous contacts between mitochondria and the smooth endoplasmic reticulum were visible within the cytoplasm (Figure 2A,B and Figure 3A). Dark supporting cells were observed between light pre-Sertoli cells or pre-spermatogonia within the seminiferous tubules in all three stages of development (Figure 1D). They possessed foot-like cytoplasmic projections that extended to the margin of the seminiferous tubule, slightly embedded in the thin basement membrane and matrix of the inter-tubular space. Long thin cytoplasmic feet extended between two adjacent cells. In the first stage of seminiferous tubule development, most of the bodies of dark supporting cells with nuclei were located above the nucleus of light pre-Sertoli cells. In the third stage, these cells were mainly observed near the basal lamina. Narrow contacts between two thin cytoplasmic extensions of support cells could be observed (Figure 3B). In calves in this study, fibroblasts, Leydig cells, and collagen fibers were identified within the intertubular space (Figure 1C,E and Figure 3F). Fibroblasts had long, thin indented nuclei with thick heterochromatin along the margins. Scattered among the fibroblasts, a few Leydig cells could be observed as individual ovoid cells with a round or elongated electron-transparent nucleus characterized by a thin heterochromatin edge. During the first two stages of seminiferous tubule development, smooth muscle cells were not observed in the intratubular area, and Leydig cells did not contain secretory vesicles. In the third stage, these regions contained fibroblasts and high amounts of collagen, but also groups of cells with highly condensed heterochromatin in a contorted nucleus.

### 3.2. Seminiferous Tubules and Intratubular Compartment in Sexually Mature Bulls

**Figure 4:**
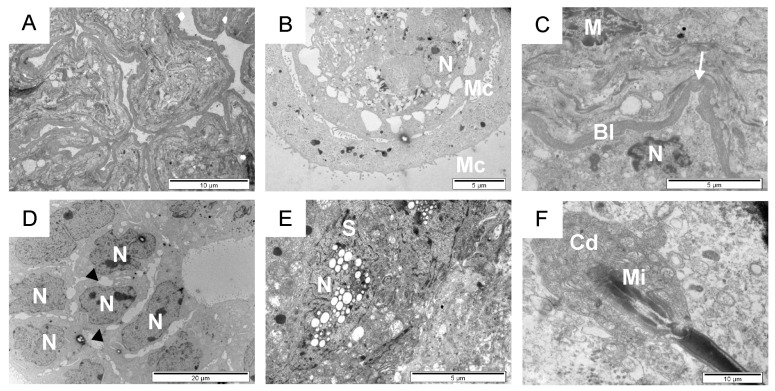
Overview of the seminiferous tubules of sexually mature bulls. Transmission electron micrographs of testicular sections (**A**–**F**). Highly contorted and columnar (**A**) structure. Simple circular and concentric (**B**) cells. Lamina propria (**C**). Spermatogonia (**D**). Sertoli cell (**E**). Sperm with the cytoplasmic droplet (**F**). Bl, tubular basal lamina; black arrowhead, large space between adjacent cells; Cd, cytoplasmic droplet; M, myofibroblast; Mc, microvilli; Mi, mitochondria; N, nucleus; S, Sertoli cells; white arrow, knob-like protrusion.

The seminiferous epithelium of sexually mature bulls is composed of spermatogonia, spermatocytes, and spermatids in various stages of development, connected intimately to Sertoli cells. Between the seminiferous tubules, in the interstitial tissue, smooth muscle cells, fibroblasts, and Leydig cells are present, closely adjacent to capillaries (Figure 4A–F). Seminiferous tubules are observed as highly contorted and columnar (Figure 4A), or as simple circular and concentric structures (Figure 4B). Germ and Sertoli cells observed within it are enclosed by a basal lamina (Figure 4C).

**Figure 5:**
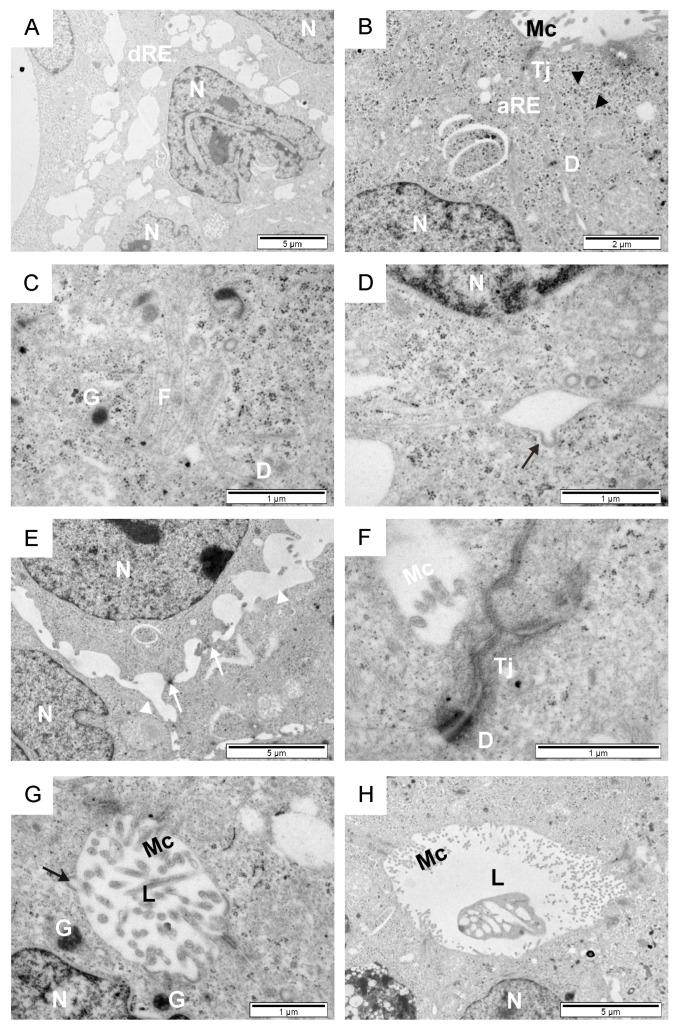
Ultrastructural appearance of spermatogonia in seminiferous tubules of sexually mature bulls (**A**–**H**). The polymorphic nuclei (**A**), annulated smooth endoplasmic reticulum (**B**), cytoplasmic filaments and glycogen in the cytoplasm (**C**), active clathrin endocytosis observed interdigitating at cell sides (**D**). Large space between adjacent cells forming arch-like membrane structures (**E**), kept in touch by cytoplasmic bridges. Cellular connections between 3 adjacent cells (**F**). Small duct which contain a thin cellular delimitation observed in the middle (**G**,**H**). aRE, annular endoplasmic reticulum; black arrow, clathrin vesicle forming; black arrowhead, free ribosomes; D, desmosome; dRE, dilated endoplasmic reticulum; F, fibres of the cytoskeleton; G, dark stained granules (glycogen); L, lumen; N, nucleus; Mc, microvilli; Tj, tight junction; white arrow, cytoplasmic bridges; white arrowheads, spaces between cells.

The basal lamina with a thickness of approx. 0.5 µm forms bulges towards the lumen of the tubule. Myofibroblasts with pear-shaped pinocytotic vesicles are visible within it on the side of the peritubular space. Spermatogonia observed in the seminiferous tubules have polymorphic nuclei with one to two nucleoli (Figure 5A). Swirls of the dilated overannulated smooth endoplasmic reticulum (sER), many cytoskeleton fibers, and glycogen granules are clearly visible in the cytoplasm (Figure 5A–C). Active endocytosis is observed in the interdigitating side of the cells (Figure 5D). Large spaces occur between neighboring cells that form arch-like membrane structures, kept in contact by cytoplasmic bridges. At the sites of the bridges, the plasmalemma is thickened by the protein density (Figure 4D and Figure 5E). On the lateral side, there are adherent junctions at about 1 µm from one cell to another. Small scarce desmosomes and 1 µm deep tight junctions are observed in the apical region (Figure 5F). Small spaces between spermatogonia, 1–2 µm in diameter, with microvilli in their narrow lumen are also visible between the cells (Figure 5G). Spermatogonia with microvilli form concentric layers of columnar or longitudinally arranged cells, delimitating a small space, 5–10 µm to 50–100 µm wide, that contains a thin cellular delimitation in the middle (Figure 4B and Figure 5G,H). In this particular area of the seminiferous tubule, there are no visible spermatids or mature spermatozoids, and the duct lumen is small and clear, with numerous microvilli protruding into the luminal space.

**Figure 6:**
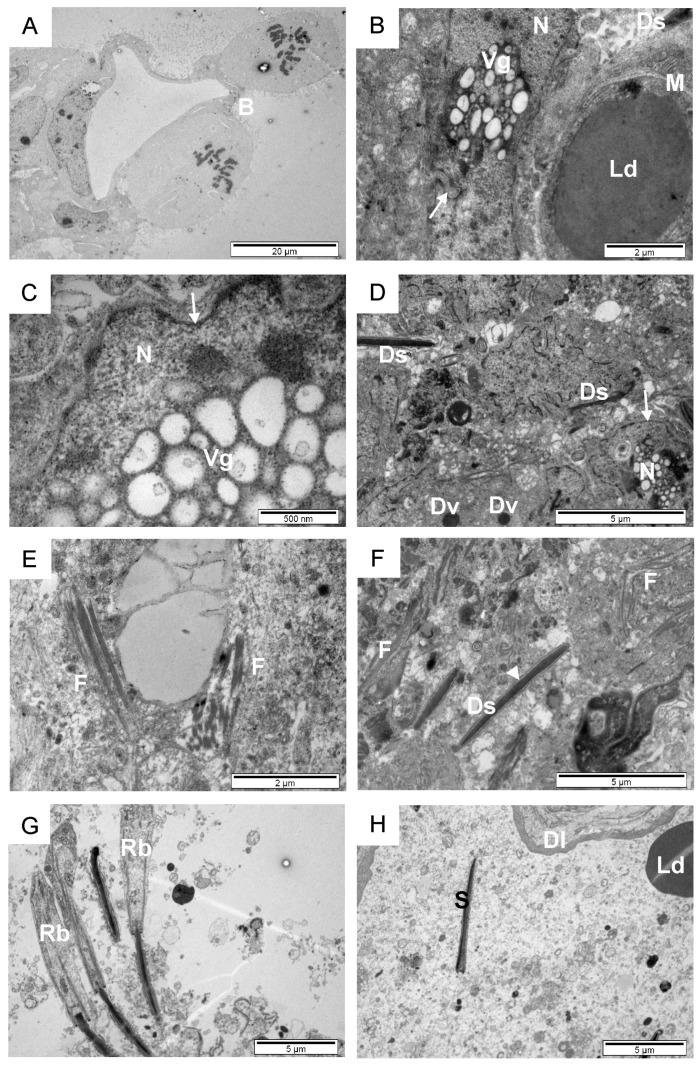
Transmission electron micrographs of the primary spermatocyte in the pro-metaphase of sexually mature bulls (**A**). Adult Sertoli cells (**B**–**D**). Bundles of cytoplasmic filaments in Sertoli cells (**E**,**F**). Spermatids with residual bodies (**G**). Lumen of seminiferous tubules (**H**). B, narrow bridge; Dl, thin electron dense layer; Ds, developing spermatid in Sertoli cell; Dv, darkly stained vesicle; F, filaments; Ld, large dark stained vesicle; N, nucleus; M, mitochondria; Rb, residual bodies; S, sperm; Vg, lightly stained vesicles group inside the nucleus; white arrow, irregular outline of the nucleus that showed numerous infoldings in its membrane; white arrowhead, junction between Sertoli cell and spermatid.

Spermatocytes in pro-metaphase can be observed in cross-sections of the testes of sexually mature bulls. A typical narrow bridge of karyoplasm is clearly visible, which connects adjacent spermatocytes (Figure 6A). Figure 4E and Figure 6B–D present details of the structure of an adult light and dark Sertoli cell. A large, characteristic nucleus that contains many cytoplasmic invaginations in its center, and also a nucleolus with many membrane-limited vesicles and tubules, is clearly visible. Within the Sertoli cells, a large darkly stained (possibly acrosomal) vesicle formed in the nearby cell can be observed (Figure 6B). Numerous cytoplasmic microfilaments and intermediate filaments are also visible within Sertoli cells (Figure 6E,F). These fibers form thick bundles that can be found along the length of the spermatids’ nuclei, and these cytoplasmic regions do not contain other organelles. Some of them are perpendicularly aligned with each other. Within the spermatid, a perinuclear ring is visible just posteriorly to the acrosome. Darker support cells (Figure 6D) have highly branched nuclei with vesicles or lysosome inclusions. In the lumen of the seminiferous tubules, numerous cytoplasmic vesicles floating with the spermatids and large vesicles (5 to 10 µm in diameter) can be observed (Figure 6H). The cross-section shows a thick electron dense layer similar to the basal laminae, which appears to limit the lumen. The presence of spermatids, with still-attached residual bodies, has been confirmed in this region. The residual bodies near the middle piece appear to be packed with mitochondria (Figure 6G). The formation of the axoneme is visible. The length of the sperm head is about 5 µm, of which ~4 µm covers the acrosomal cap. Spermatozoa with the cytoplasmic droplets were also observed (Figure 4F).

**Figure 7:**
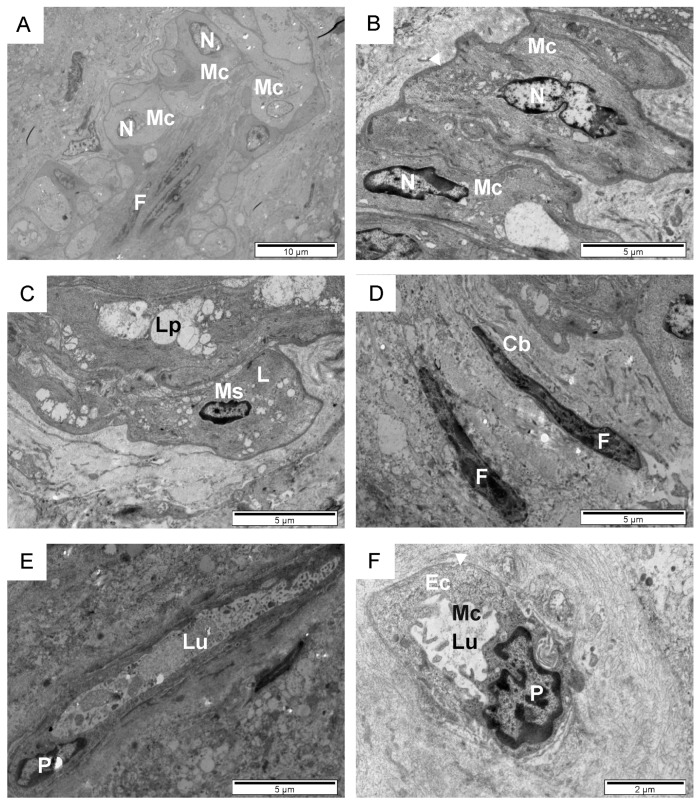
Ultrastructural appearance of testicular intertubular space of sexually mature bulls (**A**–**F**). Overview of the interstitial spaces (**A**). Smooth muscle cells (**B**,**C**). Fibroblasts (**D**). Capillaries (**E**,**F**). Cb, collagen bundles; Ec, endothelial cell; F, fibroblast; L, lysosomes; Lp, lipofuscin, Lu, lumen; Mc, microvilli; Ms, smooth muscle cells; N, nucleus; P, pericyte; white arrowhead, basal membrane.

In sexually mature animals, fibroblasts, smooth muscle cells, and capillaries are mainly observed within the space between seminiferous tubules using transmission electron microscopy (Figure 7A–D). Muscle cells form clusters of clumped interstitial cells amid a collagenous environment. Some of these cells contain lysosomes and lipofuscin deposits (Figure 7C). The observed muscle cells are characterized by polymorphic nuclei, grouped round mitochondria, large vesicles, and a thick basal membrane. Fibroblasts are long thin cells, with a furrow morphology (Figure 7A,D). They have an elongated nucleus with an average length of 15 to 20 µm and a width of 0.5 to 1 µm. Thin cell processes are also visible. In some places of this collagenous area, usually near a group of smooth muscle cells or near Leydig cells, narrow (3 × 6 µm^2^) or oval capillaries appear, with microvilli in the lumen (Figure 7E,F). Sometimes, a pericyte, which completely surrounds the endothelial cell from the basal side, and a thick basement membrane can be seen.

## 4. Discussion

In 1961, the then graduate student, R.G. Fossland, along with his teacher, the associate professor of dairy husbandry, A.B. Schultz, published the first work on the analysis of the structure of the bovine testis during postnatal development [9]. Their proposed classification into four developmental stages remains a subject of limited discussion to this day. Thus far, there has also not been much new information in this area regarding cattle. According to the classification by Fossland and Schultz [9], the calves in the present experiment belonged to the second age group of animals from the 15th to the 28th weeks of age, referred to as the prepubertal stage. During this period, the cited authors observed several layers of cell lining the tubule, including pre-spermatogonia, pre-Sertoli cells, and primary spermatocytes, as well as an increase in the number of Leydig cells in the interstitium. They also noted progressing lumenization of the seminiferous tubule and an increase in its diameter. The results obtained in this study generally corresponded to the observations of both Fossland and Schultz [9] and other authors [6,8,10,11,13,18,19,20,21]. Nonetheless, our observations complement the knowledge and provide new data regarding testicular development in cattle. The identification of three developmental stages within the seminiferous tubules indicates dynamic changes occurring in the testes of the calves examined. The observed increase in the seminiferous tubule diameter to 100 µm, as well as the thickening of the basal lamina up to 6 µm, and changes in the shape and type of cells lining the tubule, their arrangement, and distribution relative to the basal lamina were typical for this period and indicated an early onset of puberty.

In sexually mature bulls, cells belonging to all of the spermatogenesis steps were observed, situated on a thin, multilayered basal lamina, which forms characteristic folds described by some authors as knob-like protrusions [8,22]. In both calves and bulls, the cytoplasm of spermatogonia is rich in a smooth endoplasmic reticulum, forming characteristic membranous swirls. The formation of such structures appears to increase the total surface area of the smooth endoplasmic reticulum. It also creates a specific microenvironment within the cell necessary for the efficient synthesis of compounds conducive to cell proliferation and differentiation.

In adult bulls, spermatogonia maintain contact with each other through numerous cytoplasmic bridges and cell junctions. Cytoplasmic bridges maintain contact between cells even during the mitotic phase. These connections form small spaces between them, with visible microvilli. It is generally known that these microscopic projections of the plasma membrane increase the surface area for diffusion and limit the increase in cell volume; they are also involved in secretion and/or absorption processes. In this study, microvilli were observed for the first time within the seminiferous tubules. In the studied area, they were present in all visible spermatogonia, but the type of these cells was not determined. It has also not been determined whether there may be any relationship between the occurrence of microvilli and the proximity of, e.g., Sertoli cells. Without a doubt, these issues are extremely interesting and require further research in this area. To date, microvilli have not been described in the literature, while the presence of cilia in the male reproductive system has been widely discussed. According to many authors, these microtubule-based organelles, observed along the male reproductive tract, such as motile sperm flagella, multiple motile cilia, and non-motile sensory cilia, coordinate numerous processes and regulate sperm function and the surrounding environment of spermatozoa [23,24,25,26].

It is generally known that three main types of cell junctions have been identified within the testis, i.e., occluding, anchoring, and communicating junctions [27,28,29]. They occur between adjacent Sertoli cells and spermatogonia, enabling crucial interactions for proper spermatogenesis through adhesive molecules [30]. The observed different types of junctional complexes among all cells within the seminiferous tubules undoubtedly ensure proper functioning and maintain the integrity of the spermatogenic epithelium.

Numerous pre-Sertoli cells were observed in the calves studied. According to many authors, undifferentiated Sertoli cells proliferate from 4 to 13–20 weeks of age, after which a decrease in their number is observed [21]. In the examined calves, changes occurring during the maturation of Sertoli cells were recorded, from a large euchromatic nucleus to a nucleus in which the formation of characteristic vesicles and tubules was observed. The cytoplasm of light pre-Sertoli cells does not contain many organelles. The numerous contacts between mitochondria and the smooth endoplasmic reticulum indicate a high energetic demand for lipid, glycolipid, and lipoprotein synthesis. We did not observe adult Sertoli cells in the calves, which was consistent with the findings of other authors. As reported by Rawlings et al. [21], the differentiation of mature Sertoli cells appears to be complete between 30 and 40 weeks of age. The initial data from the 1960s to the 1980s indicated that there may be two types of Sertoli cells in the testes, namely dark and light cells [4,31,32,33]. Among the cells lining the seminiferous tubules in the calves examined, elongated dark supporting cells extending from the base towards the lumen were also observed. They were found in all three developmental stages between pre-spermatogonia and light pre-Sertoli cells. In certain areas, the thin cytoplasm of dark supporting cells develops protrusions through which they connect with each other and form a kind of physiological communication network. Bielańska-Osuchowska and Sysa [4] observed two types of Sertoli cells, dark and light, in 14–15-month-old bulls. Dark Sertoli cells, characterized by their electron-dense cytoplasm and indistinct nucleus, were rarely observed within the bovine seminiferous tubule. Undifferentiated dark and light supporting cells were also identified in the embryonic testes of guinea pigs [31]. Dark supporting cells were characterized by densely packed granular endoplasmic reticulum. Dark Sertoli cells in turn were previously observed in the mature boar testis [32]. The author of the latter study suggested that the difference in electron density of the supporting cells was related to their varying reactivity to the absence of gonadotropin hormones. On the other hand, Bielańska-Osuchowska and Sysa [4] indicated that these are the dark Sertoli cells that produce steroids. In our previous studies using a light microscope, it was not possible to distinguish Sertoli cells [16]. However, ultrastructure analysis confirmed that two types of support cells also occur in adult reproductive bulls. These cells had large, characteristic nuclei with many cytoplasmic invaginations, nucleoli with numerous membrane-limited vesicles of various sizes and tubules, large and small dark-stained vesicles in the cytoplasm, cytoplasmic filaments, and an abundant smooth endoplasmic reticulum. As reported by Sinowatz and Amselgruber [6], the complexity of Sertoli cell nucleoli indicated their active role in cell synthesis processes. Meanwhile, numerous intermediate filaments and microtubules imply a highly dynamic cytoskeleton, allowing for changes in shape during spermatogenesis and determining the positions of cells in the spermatogenic line within the germinal epithelium [6]. In the bulls in this study, numerous developing spermatocytes were observed within the Sertoli cells, even very close to their nuclei, which might indicate their involvement in the formation and maturation of these cells.

The postnatal development of bovine Leydig cells was described in detail by Wrobel [13]. It is widely recognized that two types of Leydig cells are present in the intratubular space. One type is the fetal generation, which gradually diminishes after birth, while the other type emerges postnatally, leading to the adult population. At 8 weeks of age in calves, degenerating fetal Leydig cells are no longer observed, while by the 16th week, their new population increases nearly fivefold. During this period, mesenchyme-like cells are increasingly less visible in the tubular space, and Leydig cells increase in size and organelle content. In the intratubular area of the test calves, fibroblasts were mainly observed, while Leydig cells were rarely recorded. According to Abd-Elmaksoud [8], the interstitial tissue of adult bulls consists of either narrow strands located between two adjacent seminal tubules or large triangular and quadrangular interstices between three to four tubules. The first contains mainly capillaries, fibroblasts, and smooth muscle cells, while Leydig cells are observed sporadically. The second space contains large blood and lymphatic vessels, as well as numerous Leydig cells arranged in strands or clusters. In the bulls in the present study, mainly the first space was observed, where adult Leydig cells with euchromatic nuclei were present sporadically. In areas where Leydig cells are not evident, their role, according to some authors, may be taken over by Sertoli cells. As reported by Sinowatz and Amselgruber [5], bovine Sertoli cells, rich in a smooth endoplasmic reticulum in the basal part, envelop the neighboring spermatids and interact with them through hormone production and transformation. It is possible that in cattle, in regions lacking Leydig cells, the dark Sertoli cells play the main role in this regard.

## 5. Conclusions

The present work refers to publications from many years ago, which are extremely valuable, and the observations of their authors remain relevant to this day. This study highlights their scientific value and contributes new data necessary for a better understanding of the male reproductive processes in cattle. Understanding the structure of male reproductive organs at the ultrastructural level will undoubtedly be key to properly interpreting many observations. These findings include results on three developmental stages of the seminiferous tubules in calves. Interestingly, in adult bulls, microvilli were found within selected cells. It appears that it should be widely acknowledged that in cattle, there are two types of Sertoli cells, which differ from each other and may have different functions. The search for markers that not only would allow the identification of Sertoli cells (such as GATA-4) [16] but also distinguish their types seems highly justified and, in the future, could prove to be very helpful in further studies. Hence, the further advancement of science and deepening of our understanding of the ultrastructure of male reproductive organs in cattle are essential measures to be undertaken.

## Data Availability

The dataset which supports the findings from this study is freely available at: Michałek, K. M., Grabowska, M., Oberska, P., Gączarzewicz, D., Syczewski, A., Tripon, S. C., Barbu, L., & Suciu, M. (2024). Ultrastructure of the bovine testis in cattle (Bos Taurus) (1–) [dataset]. Gdańsk University of Technology. https://doi.org/10.34808/q0x2-j914.

## References

[1] Michałek K., Oberska P. (2023). Aquaporins in the Male Reproductive System: A Chance for Paternity or a Road to Nowhere?. Andrology.

[2] Michalek K., Oberska P., Malkowska P., Bartkiene E. (2021). In Search of New Potential Markers for Male Fertility and Semen Quality Control. Aquaporins in Reproductive System and Metabolomic Profiling of Semen. J. Physiol. Pharmacol..

[3] Santos J.E., Thatcher W.W., Chebel R.C., Cerri R.L., Galvão K.N. (2004). The effect of embryonic death rates in cattle on the efficacy of estrus synchronization programs. Anim. Reprod. Sci..

[4] Bielańska-Osuchowska Z., Sysa P.S. (1981). Ultrastructure of the Bull Seminiferous Tubule Supporting Cells with Special Consideration of the Cell Junctions. Anat. Histol. Embryol..

[5] Goyal H.O. (1985). Morphology of the Bovine Epididymis. Am. J. Anat..

[6] Sinowatz F., Amselgruber W. (1988). Ultrastructure of Sustentacular (Sertoli) Cells in the Bovine Testis. Acta Anat..

[7] Müller E., Rodriguez-Martinez H., Braden S., Edqvist L.E. (1992). Testicular Ultrastructure of Zebu Bulls in Costa Rica. Zentralbl. Veterinarmed. A.

[8] Abd-Elmaksoud A. (2005). Morphological, Glycohistochemical, and Immunohistochemical Studies on the Embryonic and Adult Bovine Testis. Doctoral Dissertation.

[9] Fossland G.R., Schultze A.B. (1961). A Histological Study of the Postnatal Development of the Bovine Testis. Research Bulletin: Bulletin of the Agricultural Experiment Station of Nebraska No. 199.

[10] Curtis S.K., Amann R.P. (1981). Testicular Development and Establishment of Spermatogenesis in Holstein Bulls. J. Anim. Sci..

[11] Aponte P.M., de Rooij D.G., Bastidas P. (2005). Testicular Development in Brahman Bulls. Theriogenology.

[12] Oatley J.M., Reeves J.J., McLean D.J. (2005). Establishment of Spermatogenesis in Neonatal Bovine Testicular Tissue Following Ectopic Xenografting Varies with Donor Age. Biol. Reprod..

[13] Wrobel K.H. (1990). The Postnatal Development of the Bovine Leydig Cells Population. Reprod. Dom. Anim..

[14] Wrobel K.H. (2000). Prespermatogenesis and Spermatogoniogenesis in the Bovine Testis. Anat. Embryol..

[15] Wrobel K.H. (2001). Morphogenesis of the Bovine Rete Testis: Extratesticular Rete, Mesonephros and Establishment of the Definitive Urogenital Junction. Anat. Embryol..

[16] Oberska P., Grabowska M., Marynowska M., Murawski M., Gączarzewicz D., Syczewski A., Michałek K. (2024). Cellular Distribution of Aquaporin 3, 7 and 9 in the Male Reproductive System: A Lesson from Bovine Study (*Bos taurus*). Int. J. Mol. Sci..

[17] Suciu M., Mirescu C., Crăciunescu I., Macavei S.G., Leoștean C., Ştefan R., Olar L.E., Tripon S.-C., Ciorîță A., Barbu-Tudoran L. (2021). In Vivo Distribution of Poly(ethylene glycol) Functionalized Iron Oxide Nanoclusters: An Ultrastructural Study. Nanomaterials.

[18] Wrobel K.H., Schilling E., Zwack M. (1986). Postnatal Development of the Connexion Between Tubulus Seminiferous and Tubulus Rectus in the Bovine Testis. Cell Tissue Res..

[19] Moura A.A., Erickson B.H. (2001). Testicular Development, Histology, and Hormone Profiles in Three Yearling Angus Bulls with Spermatogenic Arrest. Theriogenology.

[20] Fujihara M., Kim S.M., Minami N., Yamada M., Imai H. (2011). Characterization and in Vitro Culture of Male Germ Cells from Developing Bovine Testis. J. Reprod. Dev..

[21] Rawlings N., Evans A.C., Chandolia R.K., Bagu E.T. (2008). Sexual Maturation in the Bull. Reprod. Domest. Anim..

[22] Staub C., Johnson L. (2018). Review: Spermatogenesis in the Bull. Animal.

[23] Nygaard M.B., Almstrup K., Lindbæk L., Christensen S.T., Svingen T. (2015). Cell Context-specific Expression of Primary Cilia in the Human Testis and Ciliary Coordination of Hedgehog Signalling in Mouse Leydig Cells. Sci. Rep..

[24] Girardet L., Augière C., Asselin M.P., Belleannée C. (2019). Primary Cilia: Biosensors of the Male Reproductive Tract. Andrology.

[25] López-Jiménez P., Pérez-Martín S., Hidalgo I., García-Gonzalo F.R., Page J., Gómez R. (2022). The Male Mouse Meiotic Cilium Emanates from the Mother Centriole at Zygotene Prior to Centrosome Duplication. Cells.

[26] Xie H., Wang X., Jin M., Li L., Zhu J., Kang Y., Chen Z., Sun Y., Zhao C. (2022). Cilia Regulate Meiotic Recombination in Zebrafish. J. Mol. Cell. Biol..

[27] Mruk D.D., Cheng C.Y. (2010). Tight Junctions in the Testis: New Perspectives. Philos. Trans. R. Soc. Lond. B Biol. Sci..

[28] Kopera I.A., Bilinska B., Cheng C.Y., Mruk D.D. (2010). Sertoli-germ Cell Junctions in the Testis: A Review of Recent Data. Philos. Trans. R. Soc. Lond. B Biol. Sci..

[29] Ahmed N., Yang P., Chen H., Ujjan I.A., Haseeb A., Wang L., Soomro F., Faraz S., Sahito B., Ali W. (2018). Characterization of Inter-Sertoli Cell Tight and Gap Junctions in the Testis of Turtle: Protect the Developing Germ Cells from an Immune Response. Microb. Pathog..

[30] Lui W.Y., Lee W.M. (2008). Mechanisms of Reorganization of Cell-Cell Junctions in the Testis. Front. Biosci..

[31] Black V.H., Christensen K. (1969). Differentiation in Interstitial Cells and Sertoli Cells in Fetal Guinea Pig Testes. Am. J. Anat..

[32] Chevalier M. (1979). Sertoli Cell Ultrastructure. II. Morphological Effects of Hypophysectomy in Pubescent Pigs. Ann. Biol. Anim. Bioch. Biophys..

[33] Azmi T.I., Bongso T.A., Harisah M., Basrur P.K. (1990). The Sertoli Cell of the Water Buffalo—An Electron Microscopic Study. Can. J. Vet. Res..

